# Genetic Mapping in Papillon-Lefèvre Syndrome: A Report of Two Cases

**DOI:** 10.1155/2013/404120

**Published:** 2013-11-04

**Authors:** Kaustubh Suresh Thakare, M. L. Bhongade, Pretti Charde, Shweta Kale, Priyanka Jaiswal, B. K. Somnath, Sunil Pendor

**Affiliations:** ^1^Department of Periodontology & Implantology, Sharad Pawar Dental College and Hospital, Wardha, Maharashtra 442001, India; ^2^Department of Prosthodontics, Sharad Pawar Dental College and Hospital, Wardha, Maharastra 442001, India

## Abstract

Papillon-Lefevre syndrome (PLS) is a rare autosomal recessive heterogeneous trait which is characterized by erythematous palmoplantar hyperkeratosis, early-onset periodontitis, and associated calcification of dura mater. The etiology of PLS is multifactorial with genetic, immunological, and microbial factors playing a role in etiopathogenesis. Recently identified genetic defect in PLS has been mapped to chromosome 11q14–q21, which involves mutations of cathepsin C. This paper presents a report of 2 cases of Papillon-lefevre syndrome in which diagnosis is based on clinical presentation and genetic mapping.

## 1. Introduction

Papillon-Lefèvre syndrome (PLS) is a rare autosomal recessive heterogeneous trait which was first described in 1924 by two French physicians, Papillon and Lefèvre [1]. It is characterized by erythamatous palmoplantar hyperkeratosis, early-onset periodontitis, and associated calcification of dura mater [2]. This disease usually has its onset between the ages of 1 and 4 affecting males and females equally. Its prevalence is estimated to be 1 to 4 per million in the general population with carrier rate of 2 to 4 per 1000. Consanguineous marriage is determined in 20 to 40% of patients with PLS [3].

 Cutaneous changes include well-demarcated erythematous hyperkeratotic lesions on the palms, soles, dorsum of the hands, and the interphalangeal joints. Other regions, including the eyelids, cheeks, knees, elbows, thighs, labial commissures, external malleolus, toes, and dorsal fingers may also be involved [1].

The second major feature of PLS is severe periodontitis, which starts at the age of 3 or 4 [4]. Periodontal effects appear almost immediately after tooth eruption when gingiva becomes erythematous and oedematous. The primary incisors are usually affected first and can display marked mobility by the age of 3. By the age of 4 or 5 years, all the primary teeth may have exfoliated [5]. After exfoliation, the inflammation subsides and the gingiva appears healthy. However, with the eruption of the permanent dentition, the process of gingivitis and periodontitis is usually repeated, and there is subsequent premature exfoliation of the permanent teeth at the age of 12 to 15 [6, 7]. There is a dramatic alveolar bone resorption, which leads to a “floating-in-air appearance” in the dental imaging [8].

The etiology of PLS is multifactorial with genetic, immunological, and microbial factors playing a role in etiopathogenesis. Recently identified genetic defect in PLS has been mapped to chromosome 11q14–q21, which involves mutations of cathepsin C [6, 9]. Studies in PLS patients have shown more than 90% reduction in cathepsin C activity [9]. Another important etiologic factor is an alteration of host defense owing to the decreased function of lymphocytes, polymorphonuclear leukocytes (PMNs), or monocytes [10]. Gram-negative microbial polysaccharides are generally recognized to be a primary factor in the etiology of periodontitis including periodontitis in LPS [11, 12]. The present case series report two cases of PLS in pediatric patients which are treated with multidisciplinary approach.

## 2. Presentation of Cases

### 2.1. Case 1

A 14-year-old female patient reported to the department of periodontics and implantology, in 2011, with chief complaint of teeth mobility and difficulty in mastication of 2 years due to loss of permanent teeth.

Past dental history revealed early shedding of deciduous dentition and loss of many of the permanent teeth 1 year ago due to excessive mobility. She also gave a history of thickening of the skin of the palms and soles since childhood. The rest of the family members including parents and one sibling were apparently normal.

Intraoral examination revealed that teeth numbers 13, 14, 17, 24, 27, 33, 34, 35, 37, 43, 45, and 47 were present. All the teeth were pathologically migrated. Twenty seven, 34, and 43 were grade-II mobile while the rest of the present teeth were grade III mobile. The gingiva around the teeth was inflamed, swollen, and tender while the oral mucosa covering the edentulous area appeared normal (Figures 3 and 4). Generalized plaque accumulation with halitosis was present. In addition, deep periodontal pockets and exudation were also observed. A panoramic radiograph was obtained which showed severe alveolar bone loss with about 10 to 15% bone remaining around the teeth present and impacted 28, 38, and 48 (Figure 5).

**Figure 1:**
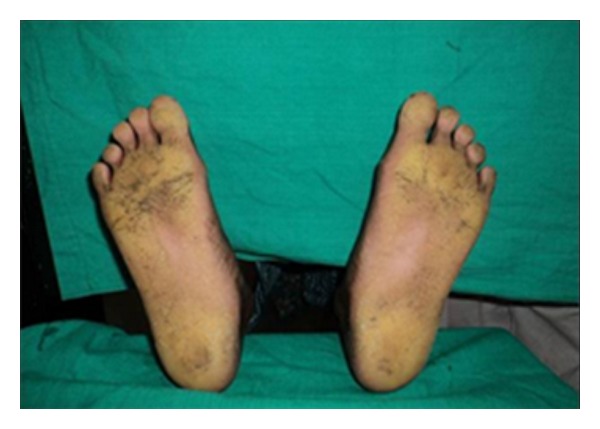
Plantar keratosis.

**Figure 2:**
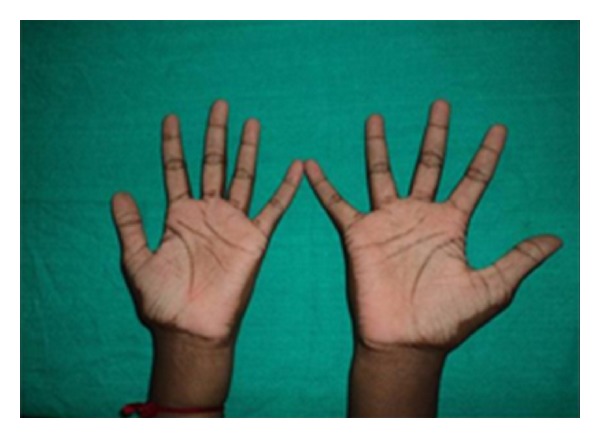
Palmar keratosis.

**Figure 3:**
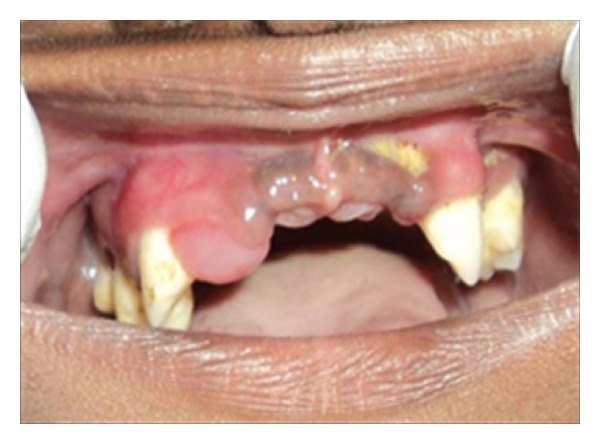
Initial clinical appearance with severe gingival inflammation and loss of several teeth.

**Figure 4:**
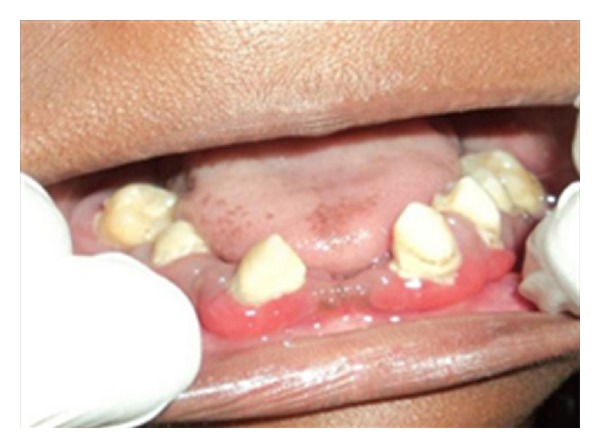
Initial clinical appearance with severe gingival inflammation and loss of several teeth.

**Figure 5:**
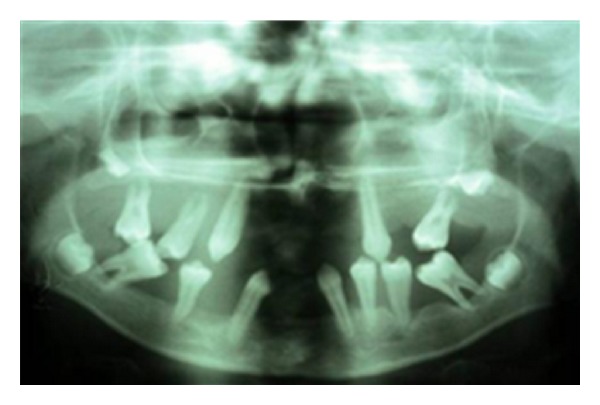
Panoramic radiograph showing “floating teeth appearance.”

Extraoral examination revealed increased keratinization of the skin of the palmar and plantar surfaces as well as the skin over the dorsal surfaces of the joints of the hands; the keratinized skin was clearly demarcated from adjacent normal skin. Deep fissures were present on the soles of her feet (Figures 1 and 2). There was associated hyperhidrosis of palms and soles resulting in a foul-smelling odor. Her nails and hair were normal.

### 2.2. Case 2

A 13-year-old male patient reported to the department of periodontics and implantology, in 2012, with chief complaint of teeth mobility, aesthetic problems, and difficulty in mastication.

The past dental history revealed that his deciduous teeth had erupted normally but exfoliated gradually by the age of 4–6. Similarly, his permanent teeth too were lost prematurely after having erupted normally.

On intraoral examination, it was found that the patient's central incisors, mandibular lateral incisors, and all permanent first molars and maxillary second molars were missing. The teeth numbers 12, 13, 14, 15, 22, 23, 24, 25, 32, 33, 34, 35, 37, 42, 43, 45, 44, 45, and 47 were present. Mobility was present in all the permanent teeth that were present. The gingiva in relation to the existing permanent teeth was red, soft, and edematous, with deep periodontal pockets and bleeding on probing. OPG of the patient showed severe alveolar bone loss in relation to the existing permanent teeth up to the level of the apical third of roots, giving the teeth a “floating in air” appearance.

On extraoral examination, there were symmetrical, well-demarcated, keratotic, and confluent plaques affecting the skin of the palms and soles, which extended to the dorsal surface of the finger joints. The skin was dry and rough on palpation. The hair and nails appeared normal.

In both cases based on the history, clinical examination, and radiographic examination, a provisional diagnosis of Papillon-Lefèvre syndrome was made. For the conformation of the diagnosis, dermatological consultation was advised and blood samples were sent for genetic mapping (Super Religare Laboratories Ltd., Nagpur, Maharashtra, India). Result of the genetic mapping revealed abnormal gene at 11q14.1–q14.3 in both cases. This gene is commonly defective in patients with Papillon-Lefèvre syndrome, which confirmed the diagnosis of the cases.

## 3. Treatment

A multidisciplinary approach is important in the management of patients with LPS. For cutaneous lesions, patients were referred to the dermatologist for dermal treatment and were treated with topical keratolytic 5% salicylic acid in combination with 10% urea and in combination with topical 8-methoxypsoralen-ultraviolet, A photochemotherapy acitretin 4 (10 mg/day) (PUVA) was initiated.

Periodontal therapy consisting of antibiotic amoxicillin (500 mg, thrice daily) and metronidazole (400 mg, thrice daily) for one week along with a mouth rinse (0.2% chlorhexidine gluconate, 10 mL twice daily) was prescribed to the patients, and the patients were educated for oral hygiene. Teeth involved with severe periodontitis were extracted and some teeth were retained for prosthetic treatment (overdenture). The patients were referred to the prosthetic department and prosthesis was planned. The patients follow-up trials were carried out and prosthetic appliances were delivered to the patient.

## 4. Discussion

Papillon-Lefèvre syndrome is a rare disorder that is inherited in an autosomal recessive manner; that is, both parents are phenotypically healthy and there is no family history of the disease, other than the affected person and possibly some siblings. Both parents must carry the autosomal gene for the syndrome to appear in their offspring. When 2 such carriers mate, there is a 25% chance of producing affected offspring [13, 14].

The exact etiology of Papillon-Lefèvre syndrome is still obscure; however, genetic, immunologic, and microbiologic factors have all been linked to the development of the syndrome. A defect of immune-mediated mechanisms including an impairment of neutrophil chemotaxis, phagocytosis, and bactericidal activities accompanied by a decrease in cell migration, reduced lymphocyte response to pathogens, depression of helper/suppressor T-cell ratio, deficient monocytic function, elevation of serum IgG, and degenerative changes of plasma cells is observed [15].

*Aggregatibacter actinomycetemcomitans *was reported to have a significant role in the progression of periodontal involvements [16]. Other microbiological agents including *Porphyromonas gingivalis, Fusobacterium nucleatum, and Treponema denticola *have also been suggested to have causal effects [17].

Recently, genetic examinations point to the Papillon-Lefèvre syndrome gene locus in these patients. The gene responsible for it has been localized to chromosome 11q14–q21 where the cathepsin C gene is, encoding a lysosomal protease, in the interval between D11S4082 and D11S931. Subsequently, inactivating mutations were identified in this gene and an almost total loss of cathepsin C activity was shown in patients with Papillon-Lefèvre syndrome. Mechanistically, deficient activation of leukocyte serine proteinases due to lack of cathepsin C gene activity could possibly explain the severe periodontitis in PLS.

PLS should be differentiated from other conditions showing similar oral or cutaneous clinical features. Diseases with oral manifestations such as acrodynia, hypophosphatasia, histiocytosis X, cyclic neutropenia, and Takahara syndrome are also associated with periodontitis and premature loss of teeth. PLS is differentiated from these other conditions by the presence of palmoplantar hyperkeratosis. PLS can also be distinguished from palmoplantar keratoderma of Unna thost, Malde Meleda, Howel-Evans syndrome, keratosis punctata, keratoderma hereditarium mutilans (Vohwinkel syndrome), and Greither syndrome as these entities are not associated with periodontal disease.

## 5. Conclusion

Even though PLS is an extremely rare condition it is associated with lifelong psychological and social impacts on the growing children such as depression including hopelessness, aimlessness, social phobia, and a fear of communicating with people outside their family. Combined cooperation from dermatologists, pediatrician, periodontists, and prosthodontists is critical for the overall care of patients suffering from PLS.
